# Risk of malignancy and necessity of completion thyroidectomy in patients with indeterminate thyroid nodules (Bethesda III and IV), more than expected in endemic region

**DOI:** 10.1007/s13304-026-02655-1

**Published:** 2026-04-29

**Authors:** Aykut Çelik, Tuğba Matlım Özel, Sezer Akbulut, Görkem Yıldız, Fatih Mert Dogukan, Serkan Sarı

**Affiliations:** 1 Division of Endocrine Surgery, Department of General Surgery, Basaksehir Cam and Sakura City Hospital, University of Health Sciences, Istanbul, Turkey; 2 Department of Pathology, Basaksehir Cam and Sakura City Hospital, University of Health Sciences, Istanbul, Turkey

**Keywords:** Completion thyroidectomy, Endemic, Fine needle aspiration biopsy, Indeterminate, Radioactive iodine therapy, Risk of malignancy

## Abstract

American Thyroid Association (ATA) argues that the prevalence of malignancy of the indeterminate nodules may vary substantially among regions, and states that it is crucial to know the prevalence of malignancy within each indeterminate cytological category at one’s institution. Our aim is to draw attention to the malignancy rates of indeterminate nodules that cannot be underestimated in an endemic region and raise awareness to differences across different populations. Between March-2021 and June-2024, 13,531 fine needle aspirations were performed on thyroid nodules in a single institution. Of these 2121 nodules were classified as indeterminate (Bethesda III–IV) and 242 patients underwent surgery. Demographic characteristics, nodule size, risk of malignancy, tumor types and subtypes were evaluated. The necessity of radioactive iodine (RAI) therapy and consequent completion thyroidectomy was investigated. Of the 242 patients 123 (50.8%) underwent lobectomy and 119 (49.2%) underwent total thyroidectomy. In total, 115 (47.5%) of 242 patients resulted in malignancy (186 patients were Bethesda-III and 82 (44.1%) of them were malignant; 56 were Bethesda-IV and 33 (58.9%) of them were malignant). Incidental carcinoma was detected in a different focus other than the indeterminate nodule in 17 patients. RAI therapy was indicated in 39 patients (33.9%) primarily based on the ATA guideline, and 24 (20.8%) patients who initially underwent lobectomy required completion thyroidectomy. Risk of malignancy in indeterminate thyroid nodules varies endemically. Each region should know their own risk and each patient’s treatment should be tailored accordingly. In this way, under-overtreatment and related morbidities will be prevented.

## Introduction

Thyroid nodules are detected in approximately 19–68% of the population and carry a 7–15% risk of malignancy (ROM) [1]. According to international guidelines, the need for further diagnostic procedures should be carefully assessed in patients with detected thyroid nodules. In this context, ultrasound-guided fine-needle aspiration cytology (FNAC) is considered the most reliable diagnostic tool [1, 2].

The Bethesda System for Reporting Thyroid Cytopathology (TBSRTC) has been utilized to classify FNAC findings based on malignancy risk. This system comprises six cytological diagnostic categories, each associated with a distinct ROM and corresponding management recommendations [3]. However, FNAC results are indeterminate (Bethesda III-IV) in approximately 10–30% of cases, malignancy rates vary across different regions and often necessitating thyroid surgery for definitive diagnosis [1, 3, 4].

In the latest edition of the TBSRTC, the ROM is reported as 13–30% for Bethesda III (Atypia of Undetermined Significance, AUS) and 23–34% for Bethesda IV (Follicular Neoplasm, FN) [3]. Other widely used international classification systems for thyroid FNAC, such as those of the UK Royal College of Pathologists (UK-RCPath), the Italian Consensus for the Classification and Reporting of Thyroid Cytology (ICCRTC), and the Japanese Reporting System for Thyroid Aspiration Cytology (JRSTAC), report varying malignancy rates for indeterminate nodules. Additionally, various studies in the literature have described significantly higher malignancy rates for these nodules [5–9].

As a general management approach, repeat FNAC or diagnostic lobectomy is recommended for Bethesda III nodules, while diagnostic lobectomy is the preferred approach for Bethesda IV nodules [1]. In this heterogeneous group, which presents challenges in management, molecular testing can be utilized to predict malignancy preoperatively. However, these tests are both expensive and not widely accessible [10–13].

Preoperative identification of malignancy in indeterminate nodules is essential, as surgeries performed for benign nodules may be considered excessive and unnecessary while also exposing patients to additional morbidity. On the other hand, inadequate treatment of malignant nodules, particularly those exceeding a certain size or belonging to aggressive subtypes, may lead to suboptimal management and necessitate repeated surgical interventions [14].

We have noticed a higher-than-expected malignancy rate among indeterminate thyroid nodules. Given that Turkey is endemic for thyroid cancer, we believe that international guidelines may not fully reflect the characteristics of local population. As stated in the American Thyroid Association (ATA) guidelines, indeterminate nodules may vary substantially among regions, and it is crucial to know the prevalence of malignancy within each indeterminate cytological category at one’s institution. Thus, we planned to evaluate and analyze the data from our own clinic.

The aim of this study is to highlight the increased malignancy rates of indeterminate nodules, which should not be underestimated in an endemic region, and to raise awareness of variations across different populations.

## Methods

Between March-2021 and June-2024, a total of 13,531 thyroid FNAC procedures performed at University of Health Sciences, Basaksehir Cam and Sakura City Hospital were reviewed. Among these, 2121 (15.7%) biopsies were classified as indeterminate (Bethesda III, AUS and Bethesda IV, FN). Surgery was recommended for all patients with FN cytology or those with two AUS results, and informed consent was obtained from all patients. Patients with an initial Bethesda III result who subsequently had benign cytology on repeat FNAC and were followed conservatively were not included to the study. Similarly, patients with repeat FNAC or concomitant FNAC from another nodule demonstrating Bethesda V–VI cytology were excluded, as they no longer represented the indeterminate group. Patients who underwent FNAC at our institution but had surgery at another center were also not included. Ultrasonographic features were not used in surgical decision-making. All data were collected prospectively and analyzed retrospectively. Procedures performed in the study were carried out in accordance with the Declaration of Helsinki, and the protocol was approved by the University of Health Sciences, Basaksehir Cam and Sakura City Hospital, Clinical Research Ethics Committee (Approval no: 2025/54).

A total of 242 patients with Bethesda III and IV cytology underwent surgery at our institution and were subsequently categorized according to the specific indeterminate cytological classification. Demographic characteristics, nodule size, type of surgical procedure, and postoperative histopathological tumor features were evaluated. The FNAC-indeterminate nodules were matched with their corresponding histopathological diagnoses in surgical specimen, and malignancy rates were calculated accordingly. Nodules diagnosed as noninvasive follicular thyroid neoplasm with papillary-like nuclear features (NIFTP) were specifically identified and considered benign.

Patients with concomitant cytological diagnoses of thyroid malignancy, hyperthyroidism, a history of prior thyroid/neck surgery or radiotherapy, those under 18 years of age, and individuals who were referred for genetic testing were not included in the study. Additionally, incidental carcinomas identified in non-indeterminate nodules was also evaluated separately and was not included in the overall malignancy rate calculations. In patients with solitary nodules, lobectomy was the preferred approach, while in those with multinodular goiter, bilateral total thyroidectomy was performed depending on the ultrasonographic features of additional nodules, family history, or patient preference.

### Management of indeterminate thyroid nodules

Thyroid nodules are evaluated by a single experienced radiologist, and FNAC is performed using a 21G needle under high-resolution ultrasound guidance with local anesthesia for nodules deemed suspicious according to the American College of Radiology Thyroid Imaging Reporting and Data System (ACR-TIRADS) [15]. Cytological preparations are examined by experienced cytopathologists and classified according to TBSRTC. For nodules diagnosed as AUS, patients are steered to re-FNAC, while lobectomy is advised for those classified as FN. If a repeat FNAC of AUS-diagnosed nodules again yields an AUS result, lobectomy is recommended for this patient group as well. Due to financial constraints, limited accessibility, and the lack of coverage by the national health insurance system, molecular testing is not considered a standard practice in Turkey. All surgeries were performed with intraoperative nerve monitoring by four general surgeons specialized in endocrine surgery, using a standardized approach under the supervision of a single mentor. Additionally, intraoperative frozen section analysis is not performed for indeterminate nodules. Specimens after surgery are sent for final histopathological examination. Necessity of radioactive iodine (RAI) therapy and completion thyroidectomy were identified in a multidisciplinary endocrine tumor board attended by endocrine surgeons, oncologists, radiologists, pathologists, and endocrinologists, all with extensive experience in thyroid cancer management. Decisions were tailored on a patient-specific basis, primarily guided by the ATA guidelines, with patients assessed within the context of intermediate- and high-risk groups, while also taking into account other current recommendations. If RAI therapy is indicated and the patient has undergone lobectomy, a completion thyroidectomy is performed before referral to nuclear medicine for RAI treatment.

### Statistical analysis

All statistical analyses were performed using SPSS software, version 27.0 (IBM Corp., Armonk, NY, USA). The distribution of continuous variables was assessed for normality using descriptive methods (Skewness–kurtosis values and coefficient of variation), graphical methods (Q-Q plots and histograms), and statistical testing (Kolmogorov–Smirnov test). Based on these evaluations, the data were determined to meet the assumptions of normal distribution. Categorical variables were presented as frequencies and percentages (n, %), while continuous variables were expressed as means and standard deviations. Comparisons between two groups for continuous variables were conducted using the independent samples t-test. For categorical variables, chi-square tests (including Pearson’s chi-square test, Fisher’s exact test, and the binomial test) were employed as appropriate. A p-value of less than 0.05 (two-sided) was considered statistically significant, with a 95% confidence interval applied throughout the analysis.

## Results

**Fig. 1:**
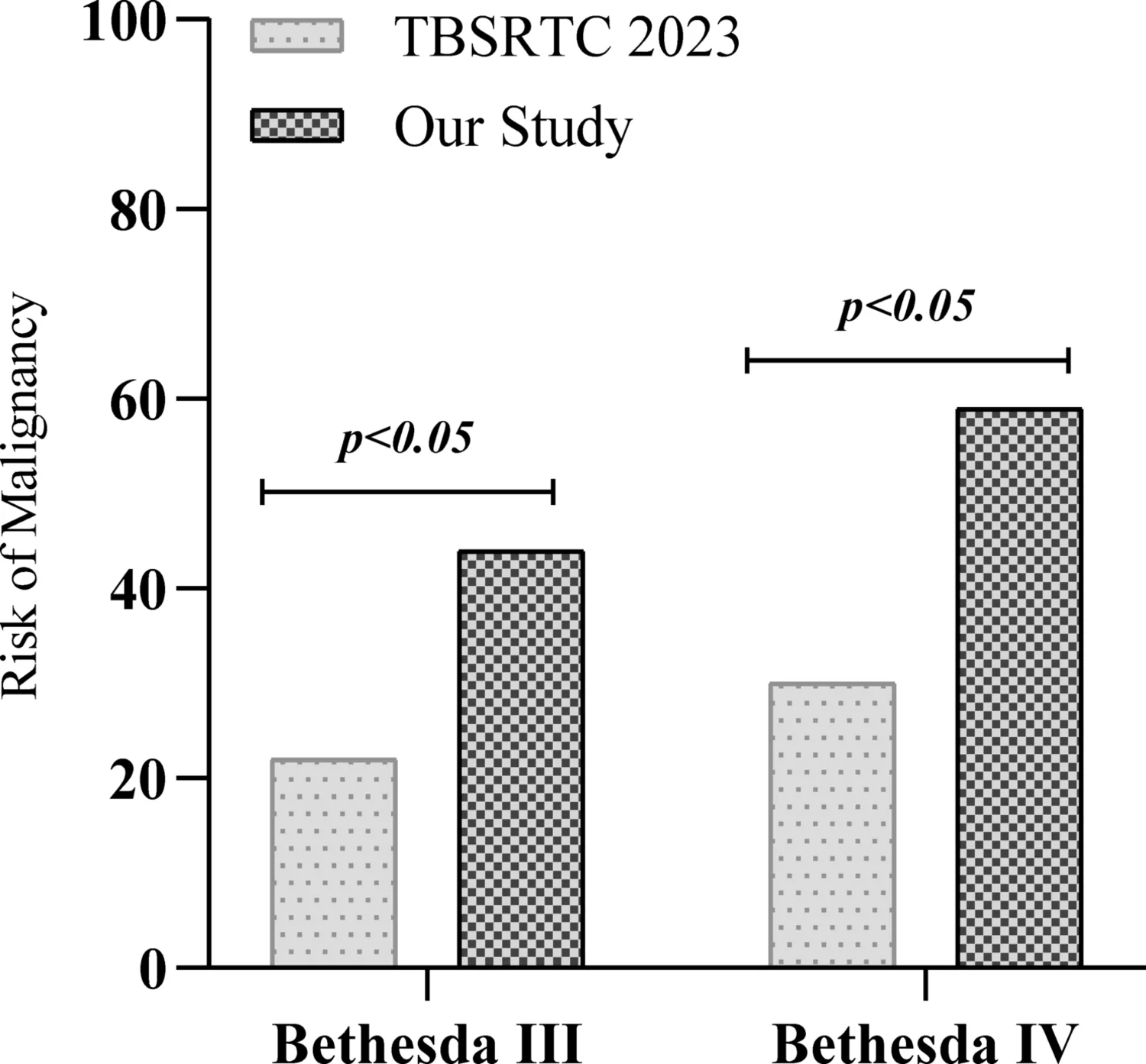
Comparison of the risk of malignancy in indeterminate thyroid nodules according to the 2023 Bethesda system for reporting thyroid cytopathology

A total of 242 patients who underwent surgery for indeterminate thyroid nodules were included in the study. The mean age was 47.7 ± 11.8 years; 188 patients (77.7%) were female and 54 (22.3%) were male. According to the Bethesda classification, 186 patients (76.9%) were categorized as Bethesda III and 56 patients (23.1%) as Bethesda IV. Postoperative histopathological analysis revealed malignancy in 115 patients (47.5%) (Table 1). When compared to the malignancy rates reported in the TBSRTC 2023, this study demonstrated significantly higher malignancy rates in both Bethesda III (n = 82; 44.1%) and Bethesda IV (n = 33; 58.9%) indeterminate thyroid nodules (Fig. 1). NIFTP was identified in 5 (2.0%) patients, while incidental carcinomas in nodules other than the indeterminate one were detected in 17 (7.0%) patients.

**Table 1:** Demographic and clinical characteristics of the patients

Variables	Bethesda III (n = 186)	Bethesda IV (n = 56)	All (n = 242)	*p*-value
Number of patients	186(76.9%)	56(23.1%)	242(100.0%)	
Age (year)^#^	47.7(11.2)	47.7(13.6)	47.7(11.8)	0.980^a^
Sex, *n(%)*				0.099^b^
Female	149(80.1%)	39(69.6%)	188(77.7%)	
Male	37(19.9%)	17(30.4%)	54(22.3%)	
Histopathology, *n(%)*				0.051^b^
Benign	104(55.9%)	23(41.1%)	127(52.5%)	
Malignant	82(44.1%)	33(58.9%)	115(47.5%)	
Nodule size (mm)^#^	26.5(15.4)	28.4(14.6)	26.9(15.2)	0.416^a^
Surgery type, *n(%)*				**0.046**^b^*
Lobectomy	88(47.3%)	35(62.5%)	123(50.8%)	
Total thyroidectomy	98(52.7%)	21(37.5%)	119(49.2%)	

*Bold indicates statistical significance, *p* < 0.05; ^#^Mean (± Standard deviation); ^a^Independent sample t test; ^b^Pearson chi-square test; ^c^Fisher’s exact test

Although the difference in malignancy rates between Bethesda III and IV was not statistically significant, a higher malignancy rate was observed in the Bethesda IV group (44.1% vs. 58.9%; p = 0.051). Notably, lobectomy was performed more frequently in patients with Bethesda IV nodules (47% vs. 63%; p = 0.046).

There were no statistically significant differences between the Bethesda III and IV groups in terms of nodule size, patient age, or sex (p > 0.05).

Among Bethesda III nodules, there was no statistically significant difference in nodule size between benign and malignant cases (p > 0.05). However, in the Bethesda IV group, malignant tumors had a significantly larger mean nodule diameter compared to benign ones (22.8 ± 10.5 mm vs. 32.3 ± 15.9 mm; p = 0.010). When applying the 4 cm cut-off value suggested in the ATA guidelines, nodule size was also significantly associated with malignancy in both the Bethesda IV group (p = 0.008) and the overall cohort (p = 0.014). No significant association was found between gender and malignancy in any group. When all indeterminate nodules were analyzed, younger age was significantly associated with an increased risk of malignancy (p < 0.001). This association was particularly evident in patients under the age of 30 within the Bethesda III group. Although the difference did not reach statistical significance in the Bethesda IV group, likely due to the smaller sample size, a trend toward higher malignancy rates in younger patients was observed (Table 2).

**Table 2:** Comparison of demographics and nodule size between benign and malignant Bethesda III and IV nodules

	Bethesda III (n = 186)			Bethesda IV (n = 56)			All (n = 242)		
Variables	Benign (n = 104)	Malignant (n = 82)	*p*-value	Benign (n = 23)	Malignant (n = 33)	*p*-value	Benign (n = 127)	Malignant (n = 115)	*p*-value
Nodule size, (mm)^#^	26.6(13.6)	26.4(17.4)	0.915^a^	22.8(10.5)	32.3(15.9)	**0.010**^a^*	25.9(13.1)	28.1(17.2)	0.283^a^
Nodule size, n(%)			0.234^b^			**0.008**^c^*			**0.014**^b*^
< 40 mm	86(58.1)	62(41.9)		22(51.2)	21(48.8)		108(56.5)	83(43.5)	
≥ 40 mm	18(47.4)	20(52.6)		1(7.7)	12(92.3)		19(37.3)	32(62.7)	
Sex,* n(%)*			0.392^b^			0.242^b^			0.917^b^
Female	81(54.4)	68(45.6)		18(46.2)	21(53.8)		99(52.7)	89(47.3)	
Male	23(62.2)	14(37.8)		5(29.4)	12(70.6)		28(51.9)	26(48.1)	
Age (year), *n(%)*			0.025*^a^			0,430^b^			< 0.001*^a^
21–30	3(21.4)	11(78.6)	0.007*^a^	1(20)	4(80)	0,639^b^	4(21.1)	15(78.9)	0.004*^a^
31–40	13(44.8)	16(55.2)	0.191^a^	7(41.2)	10(58.8)	> 0.999^b^	20(43.5)	26(56.5)	0.174^a^
41–50	51(64.6)	28(35.4)	0.041*^a^	7(58.3)	5(41.7)	0.200^b^	58(63.7)	33(36.3)	0.006*^a^
51–60	25(61)	16(39)	0.460^a^	5(50.0)	5(50)	0.725^b^	30(58.8)	21(41.2)	0.307^a^
≥ 61	12(52.2)	11(47.8)	0.700^a^	3(25.0)	9(75)	0.322^b^	15(42.9)	20(57.1)	0.218^a^

*Bold indicates statistical significance, *p* < 0.05; **#**Mean (± Standard deviation); ^a^Independent sample t test; ^b^Pearson chi-square test; ^c^Fisher’s exact test

The majority of malignant tumors were papillary carcinomas (n = 86; 74.8%) (Including invasive encapsulated follicular variant papillary carcinoma, as classified under the 2022 World Health Organization (WHO) classification). Of these, 45 cases (52.2%) were identified as microcarcinomas (< 1 cm). When comparing Bethesda categories, papillary carcinoma was significantly more frequent in the Bethesda III group (84% vs. 52%; p < 0.001), whereas oncocytic carcinoma was more commonly observed in the Bethesda IV group (7% vs. 24%; p = 0.023). Although follicular carcinoma was more frequently observed in Bethesda IV nodules, the difference did not reach statistical significance, likely due to the limited sample size. Analysis of histopathological subtypes revealed that the most common variants were the classic subtype (61.6%) in papillary carcinoma and minimally invasive subtype (71.4%) in follicular-oncocytic carcinoma (Table 3).

**Table 3:** Comparison of histopathological types and subtypes

Histopathological types,* n(%)*	Bethesda III (n = 82)	Bethesda IV (n = 33)	All (n = 115)	*p*-value
Papillary carcinoma	69(84.1%)	17(51.5%)	86(74.8%)	** < 0.001**^**b**^
Follicular carcinoma	7(8.5%)	7(21.2%)	14(12.2%)	0.110^c^
Oncocytic carcinoma	6(7.3%)	8(24.2%)	14(12.2%)	**0.023**^c^*
Poorly differentiated carcinoma	0(0.0%)	1(3.0%)	1(0.9%)	0.287^c^
Histopathological subtypes,* n(%)*				
Papillary carcinoma	(n = 69)	(n = 17)	(n = 86)	
Classic	44(63.8%)	9(52.9%)	53(61.6%)	
Infiltrative follicular	8(11.6%)	6(35.3%)	14(16.3%)	
Invasive encapsulated follicular	8(11.6%)	0(0.0%)	8(9.3%)	
Oncocytic	5(7.2%)	1(5.9%)	6(7.0%)	
Solid/trabecular	2(2.9%)	1(5.9%)	3(3.5%)	
Tall cell	2(2.9%)	0(0.0%)	2(2.3%)	
Follicular—Oncocytic carcinoma	(n = 13)	(n = 15)	(n = 28)	
Minimally invasive	9(69.2%)	11(73.3%)	20(71.4%)	
Encapsulated angioinvasive	2(15.4%)	2(13.3%)	4(14.3%)	
Widely invasive	2(15.4%)	2(13.3%)	4(14.3%)	
Other	(n = 0)	(n = 1)	(n = 1)	
Poorly differentiated carcinoma	0(0.0)	1(100%)	1(100%)	

*Bold indicates statistical significance, *p* < 0.05; ^#^Mean (± Standard deviation); ^a^Independent sample t test; ^b^Pearson chi-square test; ^c^Fisher’s exact test

When adverse histopathological tumor features were compared, the mean tumor size was significantly larger in Bethesda IV group compared to Bethesda III (31.1 ± 22.7 mm vs. 21.3 ± 21.5 mm; p = 0.032). Lymphatic invasion was more frequently observed in Bethesda III nodules (26% vs. 9%; p = 0.049), whereas vascular invasion was significantly more common in Bethesda IV nodules (9% vs. 24%; p = 0.033) (Table 4).

**Table 4:** Histopathological tumor features

Variables	Bethesda III (n = 82)	Bethesda IV (n = 33)	All (n = 115)	*p*-value
Tumor size (mm)^#^	21.3(21.5)	31.1(22.7)	24.1(22.2)	**0.032**^a^*
Encapsulated,* n(%)*	60(73.2)	26(78.8)	86(74.8)	0.530^b^
Capsular invasion,* n(%)*	53(64.6)	23(69.7)	76(66.1)	0.604^b^
Lymphatic invasion,* n(%)*	21(25.6)	3(9.1)	24(20.9)	**0.049**^b^*
Vascular invasion,* n(%)*	7(8.5)	8(24.2)	15(13.0)	**0.033**^c^*
Perineural invasion,* n(%)*	3(3.7)	0(0.0)	3(2.6)	0.556^c^
ETE,* n(%)*	0(0.0)	0(0.0)	0(0.0)	N/A
Multifocality/multicentricity,* n(%)*	37(45.1)	10(30.3)	47(40.9)	0.144^b^

*Bold indicates statistical significance, *p* < 0.05; ^#^Mean (± Standard deviation); ^a^Independent sample t test; ^b^Pearson chi-square test; ^c^Fisher’s exact test; N/A, not applicable, ETE: Extrathyroidal extension

**Fig. 2:**
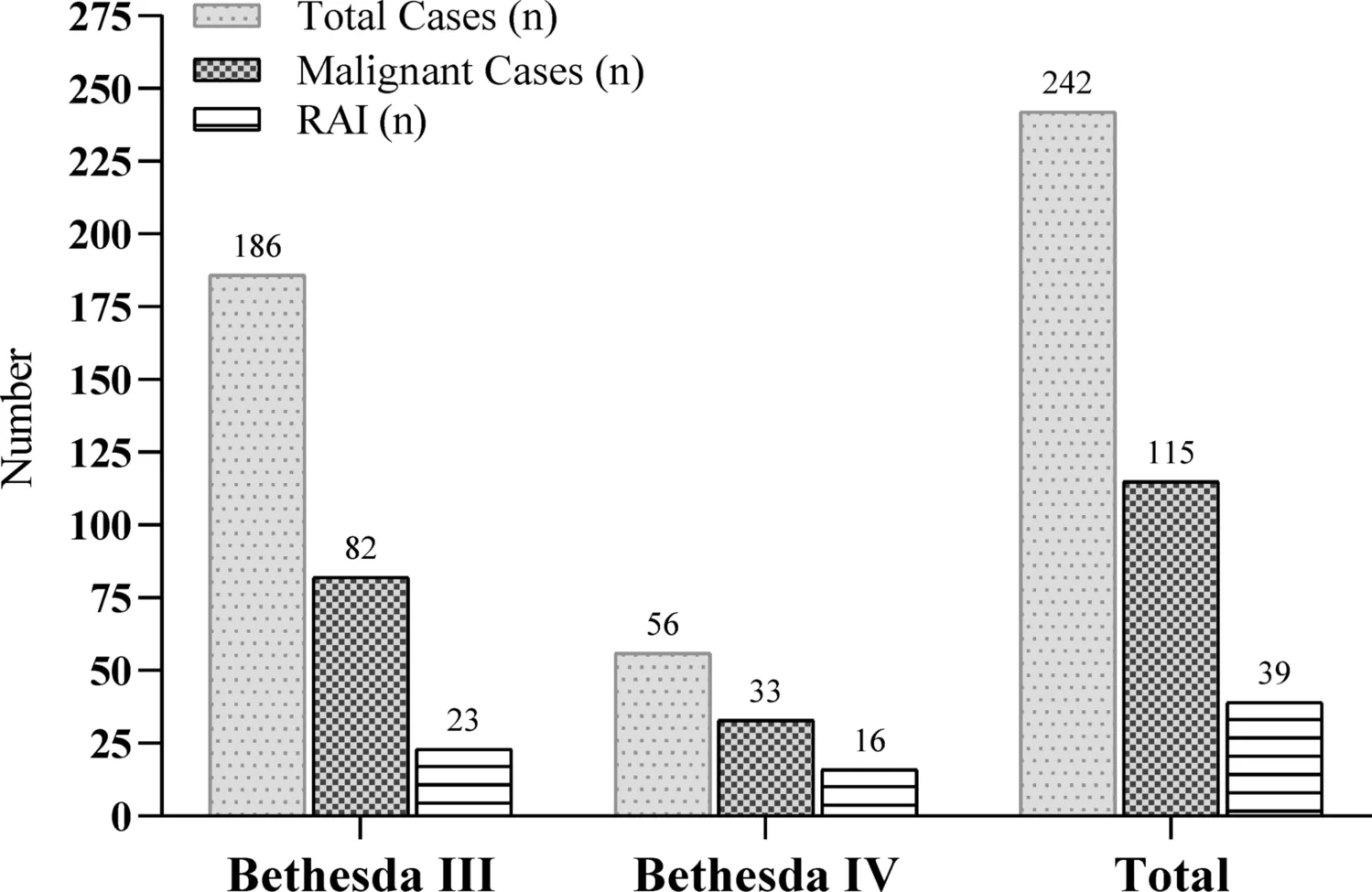
Schematic distribution of indeterminate thyroid nodules, including the number of malignant cases confirmed by final histopathology and those for whom radioactive iodine therapy was indicated

Among the 186 patients with Bethesda III cytology, 82 (44.1%) were confirmed to have malignancy on final histopathology, and 23 of these (12.3% of all; 28% of malignant Bethesda III cases) were indicated for RAI therapy. Similarly, of the 56 patients with Bethesda IV cytology, 33 (58.9%) were diagnosed with malignancy, and 16 of these (28.5% of all; 48% of malignant Bethesda IV cases) were indicated for RAI therapy. When comparing the two groups, the need for RAI therapy was significantly higher in the Bethesda IV group (p = 0.036). In total, 115 of the 242 patients with indeterminate nodules were diagnosed with malignancy, and 39 (16.1% of the overall cohort; 33.9% of malignant cases) were indicated for RAI therapy (Fig. 2). Among these, 15 patients had already undergone total thyroidectomy due to multinodular goiter, while the remaining 24 (20.8%) required completion thyroidectomy.

## Discussion

The management of indeterminate thyroid nodules has consistently posed clinical challenges. Variability in malignancy rates across different geographic regions, the use of diverse diagnostic algorithms, and the existence of multiple classification systems further complicate decision-making. Even within the globally accepted TBSRTC, substantial revisions have been introduced over time, particularly regarding the indeterminate categories. Notably, the estimated malignancy risk for Bethesda III nodules has increased from 5–15% to 13–30%, while for Bethesda IV nodules it has been revised from 15–30% to 23–34% [3, 16–18].

Various investigations have been conducted to reduce the complexity associated with the management of indeterminate thyroid nodules. Although molecular testing has gained considerable popularity in recent years for its ability to accurately stratify malignancy risk in indeterminate thyroid nodules and reduce unnecessary surgeries, its widespread use is limited by high costs and limited accessibility [11, 19, 20]. Similarly, studies investigating nuclear atypia on cytological specimens have reported that the presence of nuclear atypia in indeterminate nodules is associated with an increased risk of malignancy, a distinction that has been emphasized in the most recent 2023 revision of the Bethesda classification [21, 22]. Another layer of complexity is the terminology of NIFTP. This entity has been incorporated into most classification systems and is considered as a benign lesion, thereby lowering the overall reported malignancy rates. According to the Bethesda classification, when NIFTP cases are excluded, the malignancy risk drops to approximately 16% for Bethesda III and 23% for Bethesda IV nodules. However, the prevalence of NIFTP also varies between populations. It is reported to be rare in Asian populations (1.6%) but considerably more frequent in non-Asian populations (up to 13.3%) [23]. This geographic variability further contributes to differences in reported malignancy rates.

The most commonly used cytopathology classification systems worldwide include TBSRTC, the UK-RCPath, the ICCRTC, and the JRSTAC. Although these systems differ in terminology and subclassification, they define indeterminate thyroid nodules in broadly comparable ways. In TBSRTC, indeterminate categories include Bethesda III, characterized by limited nuclear or architectural atypia insufficient for definitive diagnosis, and Bethesda IV, which is primarily based on a microfollicular architectural pattern with scant colloid. The UK-RCPath system similarly distinguishes Thy3a (atypia of undetermined significance, often with focal nuclear or architectural atypia) from Thy3f (lesions with features suggestive of follicular neoplasm). The ICCRTC further refines this group by subdividing indeterminate lesions into low-risk (TIR3A) and high-risk (TIR3B) categories, placing greater emphasis on the degree of cellularity, architectural atypia, and subtle nuclear features to improve risk stratification. In contrast, the JRSTAC system incorporates cytological findings alongside clinical and ultrasonographic features and may apply different thresholds for surgical recommendation, particularly in follicular-patterned lesions. Despite these differences, all systems rely on similar cytomorphological features, including varying degrees of nuclear atypia, architectural atypia (particularly microfollicular patterns), and reduced colloid, which are insufficient for a definitive benign or malignant diagnosis. However, the importance assigned to these features differs among systems; some place greater emphasis on nuclear atypia, while others prioritize architectural patterns. In addition, the threshold for how much atypia is considered acceptable within the indeterminate category varies, leading to differences in classification and risk stratification. Consequently, all guidelines acknowledge substantial heterogeneity in malignancy risk within these categories, with reported rates ranging widely from approximately 12% to 55% [5, 6, 9, 24].

ATA guidelines suggest that malignancy risk increases with nodule size. However, as noted by Trimboli et al., this association remains a matter of debate for indeterminate nodules [25]. Studies by Valderrabano P. et al. and Cozzani F. et al. did not identify a significant correlation between nodule size and malignancy in indeterminate nodules [26, 27]. We similarly found no statistically significant association between nodule size and malignancy in Bethesda III nodules. However, in Bethesda IV nodules, larger nodule size appeared to be statistically associated with a higher ROM. These findings are consistent with those of Macias A. et al., who also reported a significant correlation between nodule size and malignancy specifically in Bethesda IV nodules [28].

Young age and male sex are known to be associated with an increased ROM in thyroid nodules [29–32]. While some studies have extended this association to indeterminate nodules, reporting that younger patients and males carry a higher malignancy risk [33], others have found no significant relationship between these variables and malignancy in this subgroup [27, 34, 35]. In this study, younger age was significantly associated with malignancy in indeterminate nodules, particularly in the Bethesda III group. Although no statistically significant association was observed in the Bethesda IV group, probably due to the smaller sample size, we believe that younger age may still be clinically relevant in predicting malignancy in Bethesda IV nodules. No statistically significant difference in malignancy risk was identified between male and female patients.

Consistent with the literature, the most common final histopathological diagnosis following surgery was papillary thyroid carcinoma in this study [29, 30, 33, 36]. NIFTP was identified in 5 (2.0%) patients, while incidental carcinomas in nodules other than the indeterminate one were detected in 17 (7.0%) patients. Based on postoperative histopathological results, 39 patients (33.9%) were indicated for RAI therapy following evaluation in a multidisciplinary endocrine tumor board. Although current guidelines recommend lobectomy for indeterminate nodules, our findings highlight that in an endemic region like Turkey, approximately one in three patients with an indeterminate nodule may eventually require RAI therapy and often necessitating a second surgical intervention for completion thyroidectomy. In this study, it was revealed that completion thyroidectomy was necessary in 24 (20.8%) patients who had initially undergone lobectomy. This represents a notably high rate and underscores the importance of population-specific management strategies.

In a comparative review conducted by Inabnet B. et al., data from four endocrine surgery registries (the Collaborative Endocrine Surgery Quality Improvement Program (CESQIP, North America), Eurocrine (Europe), the Swedish Quality Registry for Thyroid, Parathyroid and Adrenal Surgery (SQRTPA, Sweden), and the United Kingdom Registry of Endocrine and Thyroid Surgery (UKRETS, UK)) were analyzed across two continents. The study reported a considerable variation in the ROM for indeterminate thyroid nodules. For Bethesda III nodules, the average ROM was found to be 36.8% in the CESQIP, compared to 25.3% in the Eurocrine registry. Similarly, for Bethesda IV nodules, ROM was 41.3% in CESQIP and 26.1% in the UK registry. Although this large-scale analysis does not encompass global data, it clearly demonstrates that regional differences in malignancy risk are substantial and should not be overlooked. The same study also addressed age-related variability in ROM, reporting higher malignancy rates in younger patients with ROM values ranging from 39.2% to 72.6% for Bethesda III nodules and from 28.1% to 71.9% for Bethesda IV nodules. Additionally, the study also noted that the Eurocrine and UK cohorts showed more similar outcomes, a finding the authors attributed to potential geographic proximity and shared regional characteristics [30].

In a large-scale study utilizing the American NSQIP database, Delman A.M. et al. reported a risk of malignancy of 36.2% for Bethesda III and 36.7% for Bethesda IV nodules. They also identified male sex and younger age as factors significantly associated with malignancy. However, as noted in the study’s limitations, the database’s inherent heterogeneity poses a challenge, and the biopsied nodule may not always correspond precisely to the malignant nodule identified in the final pathology. Furthermore, the study did not exclude cases of NIFTP or those who underwent molecular testing, potentially influencing the overall malignancy rates [33].

Studies comparing Eastern and Western populations have reported higher malignancy risks in Asian cohorts, with this difference being particularly pronounced in indeterminate thyroid nodules. Notably, research originating from endemic regions such as South Korea has consistently demonstrated malignancy rates that exceed the estimates provided by the TBSRTC. However, these studies have also acknowledged limitations, including selection bias and heterogeneity within study groups, which may have influenced the reported outcomes. [8, 37, 38].

More studies have reported malignancy risks exceeding 50% for indeterminate thyroid nodules. However, most of these studies exhibit notable methodological limitations. In many cases, selection bias arises as a result of stratifying patients based on molecular testing or ultrasound features, with some patients being followed conservatively rather than undergoing surgery. Additionally, in patients with multinodular goiter, incidental carcinomas arising from nodules other than the one sampled by FNAC are often not separately analyzed and are instead included in the overall malignancy rate. Furthermore, some studies have not classified NIFTP, potentially inflating malignancy estimates. Due to these methodological inconsistencies, it is difficult to establish a precise and universally applicable ROM for indeterminate nodules [5, 7, 22, 29, 36, 37, 39].

An important methodological feature of this study is that all patients evaluated in our clinic who had FN or two repeated AUS FNAC results were referred for surgery regardless of ultrasound findings, and their data were collected prospectively. Patients who underwent molecular testing were not included in the study, thereby minimizing the potential for selection bias. Additionally, each nodule sampled by FNAC was carefully matched with the corresponding nodule identified during postoperative histopathological examination to ensure accurate malignancy rate calculations. Incidental carcinomas detected in nodules other than the one biopsied were not included in the malignancy rate. As a limitation, this study reflects data from a single tertiary center. Therefore, the findings may not be generalizable to the broader population, both in terms of sample size and geographic representation.

Why is thyroid cancer disproportionately more common in our population? According to the 2024 American Cancer Society data, thyroid cancer does not rank among the top ten most common estimated new cancer cases in men in the United States, whereas it ranks eighth among women [40]. Globally, the WHO reports thyroid cancer as the seventh most commonly diagnosed cancer, yet it ranks only 24th in cancer-related mortality, with a significantly higher incidence observed in Asian populations [41]. In contrast, national cancer statistics from Turkey reveal a strikingly higher burden: thyroid cancer ranks eighth among men and second among women, following breast cancer. Alarmingly, in females under the age of 25, thyroid cancer is the most frequently diagnosed malignancy [42]. Turkey is characterized by a predominantly young population, and the average age in our study was lower than that reported in similar studies from other regions, which may potentially account for the higher observed malignancy rates. Moreover, iodine deficiency is a well-recognized risk factor for thyroid cancer and remains a relevant public health concern in Turkey. Although iodization of table salt has been mandatory since a nationwide policy was implemented in 1998, iodine deficiency still persists in certain regions. Additionally, despite the decades that have passed since the incident, several studies suggest that the 1986 Chernobyl nuclear disaster may still be contributing to an elevated risk of thyroid cancer in Turkey, particularly in the northern regions [43–45].

## Conclusion

The risk of malignancy in indeterminate thyroid nodules is known to vary significantly across different geographic regions, particularly in areas with endemic characteristics. Therefore, it is essential for each institution and region to determine and be aware of their own malignancy rates based on local data. Tailoring treatment decisions to both regional risk profiles and individual patient characteristics is crucial to ensure appropriate management. Such a personalized and region-specific approach may help avoid both overtreatment and undertreatment, thereby reducing the likelihood of related morbidities and unnecessary surgical interventions.

## Data Availability

All data used in this study were stored in a Microsoft Excel file.
